# Motor imagery, forward models and the cerebellum: a commentary on Rieger et al., 2023

**DOI:** 10.1007/s00426-023-01916-7

**Published:** 2024-01-18

**Authors:** R. C. Miall

**Affiliations:** School of Psychology, University of Birmingham, Birmingham, UK

## Abstract

In this commentary on Rieger et al., Psychological Research Psychologische Forschung, 2023, I discuss possible ways to test the hypothesis that action imagery is achieved by simulations of actions through an internal forward model. These include brain imaging, perturbation through TMS, and psychophysical tests of adaptation of intended reach actions.

Rieger and colleagues (2023) make a convincing argument for the role of internal models operating in action imagery, and specifically forward models being the mechanism through which actions are simulated, and through which errors in imagery are detected. This fits well with the general concept of forward internal models in motor control (Miall & Wolpert, 1996), neural representations that are understood to receive efference copy of motor commands, as well as sensory inputs and that then generate an internal estimate of the sensory consequences of those commands (Wolpert et al., 1998). As the output is a sensory representation (or an estimate of the internal state of the motor system, that can be converted to a sensory representation), the forward model output could be available as an imagined representation of action. During imagery, no motor commands should reach the musculature, or else movement would be generated. Hence, the internal efferent copies must be either derived independently of descending commands, or the commands inhibited downstream of the internal model. Errors in action imagery are occasionally generated, with the implication that mistakes are made either in the generation of the command or efference copy, relative to the desired action, or in the forward model estimation based on this command, or possibly in the comparison process. Finally, there are possible alternatives to motor system-based simulation of actions, particularly the use of generalized knowledge, possibly gained through observation of one’s own or others’ actions.

These basic facts (as laid out in greater detail by Rieger et al.) suggest that their hypothesis could be tested by challenging the forward model.

First, it is likely that the cerebellum performs forward model operations and is potentially the main—if not exclusive—site of a motor-related forward model (Miall et al., 1993; Sokolov et al., 2017; Wolpert et al., 1998). It receives descending motor commands and its outputs project back to fronto-parietal cortical areas that might subserve mental imagery. Hence, one could test for cerebellar activation during mental imagery, and indeed many studies have done so. Hétu et al. (2013) provide a meta-analysis of evidence from 75 brain imaging papers, and the cerebellum is consistently activated, with ipsilateral loci consistent with upper and lower limb actions. However, imaging studies alone cannot exclude a cerebellar contribution in other ways, independent of forward modeling—for example in the inverse model responsible for motor command generation.

Second, one could look for a causal relationship, by testing mental imagery during disturbances of cerebellar function. Battaglia et al. (2006) found that stroke affecting the cerebellum disrupted the changes in excitability of motor cortex that are normally induced by motor imagery. More directly, González et al. (2005) found that cerebellar stroke survivors showed slowing of finger sequences in both actual and imagined conditions, consistent with the cerebellum contributing to mental simulation. There are few reports of direct modulation of the cerebellum by transcranial magnetic or electrical stimulation, although Grami et al. (2022) and Cengiz and Boran (2016) have independently found that TDCS of the cerebellum influenced the extent that imagery of actions could modulate cerebral cortical activity, analogous to Battaglia et al.’s (2006) result. However, we previously reported that cerebellar TMS can selectively bias reaching movements to a visual target, and this shift in reach direction is likely to be because of the temporary blockade of cerebellar forward model output (Miall et al., 2007). This task would be well suited to test mental imagery: the final position of the hand could be reported relative to the target for imagined reaches with and without TMS. I would predict a directional bias in imagined hand end-point during stimulated trials, as is seen in active movement, and this would be strong evidence for the forward model operating during action imagery.

Third, one might explore the issue of forward model corrections and learning during imagined action errors. It is well documented that imagining and rehearsal of actions can lead to improved performance, and the assumption is that success in the imagined action leads to beneficial changes in execution. Rieger et al. (2023) discuss the converse situation and report imagined errors, albeit at a frequency lower than in actual actions. It seems plausible that errors might accumulate throughout the neural chain of command, from planning of intended actions, the generation of commands, prediction of their consequences, in the integration of the commands with brainstem and spinal circuit activity, or in muscular execution. Because there are no action outcomes in imagery, perception of errors in imagined actions is probably only possible if they occur in the first three stages, or in the comparison of intention and imagined outcome. One can ask what changes these errors lead to—is it in the intended actions or the forward modeling of their consequences? Recent papers (Morehead et al., 2017; Tsay et al., 2022) have shown that presentation of a “visually clamped” error after a reaching action, regardless of the actual hand reach direction, leads to the implicit adaptation of the reach movements, to compensate for the sensory prediction error between action and displayed feedback. These sensory prediction errors drive cerebellar-dependent learning (Tseng et al., 2007). One could perform an experiment with imagined reach actions toward a visual target with a visual “error” presented after each imagined action that is clamped to one side of the target. I would predict that the imagined reaches would gradually adapt as do actual actions (Tsay et al., 2022). After adaptation, one could test the intended action, the imagined outcome, and the direction of actual reaches, to separate out where the adaptive changes have occurred. If the forward model was adapted because of these errors in imagery, then there should be a remapping of both intended action and visual outcome, but if only the intentions are adapted, there would be no remapping, only a shift in intended reach direction.

Finally, Rieger et al. (2023) discuss the relationship between imagery through action simulation (i.e., forward models) versus propositional knowledge of actions. It is interesting to explore the experience-dependent nature of imagery: forward models are developed through experience of actions, with a sensory prediction error between expected and actual outcome driving their improved accuracy (Tseng et al., 2007). One approach would be to test action imagery in the absence of recent experience of certain actions. Chronic or congenital loss of limbs is an obvious choice, and Malouin et al. (2009) report reduced vividness of imagined actions after amputation or disuse of a limb. Congenital or chronic loss of sensation is also likely to lead to a degraded forward model process, and IW, a man with 4 decades of profound somatosensory loss, has degraded kinaesthetic motor imagery, while still having intact or enhanced visual imagery (ter Horst et al., 2012). While not directly implicating the cerebellum, these results are consistent with a motor simulation degraded because of the chronic absence of sensory inflow to the forward model.

## Data Availability

Not available.
